# In situ MALDI mass spectrometry imaging of pharmaceuticals and metabolites in human teeth and jawbone

**DOI:** 10.1007/s00216-026-06515-0

**Published:** 2026-04-23

**Authors:** Hameda Amin, Marcus Daniel Brandbjerg Bohn Lorensen, Harald Severin Hansen, Azam Bakhshandeh, Thomas Kofod, Sanne Werner Moeller Andersen, Camilla Kragelund, Christian Janfelt

**Affiliations:** 1 Department of Pharmacy, Faculty of Health and Medical Sciences, University of Copenhagen, Universitetsparken 2, 2100 Copenhagen, Denmark; 2 Department of Drug Design and Pharmacology, Faculty of Health and Medical Sciences, University of Copenhagen, Universitetsparken 2, 2100 Copenhagen, Denmark; 3 Faculty of Odontology, Department of Oral Pathology, Malmö University, Malmö, Sweden; 4 Department of Oral & Maxillofacial Surgery, Copenhagen University Hospital (Rigshospitalet), Copenhagen, Denmark; 5 Department of Odontology, Faculty of Health and Medical Sciences, University of Copenhagen, Nørre Alle 20, 2200 Copenhagen, Denmark

**Keywords:** MALDI-MSI, Mass spectrometry imaging, Teeth, Jawbone, Analgesics, Chlorhexidine, MRONJ, Pharmaceuticals

## Abstract

Teeth and jawbone are mineralized tissues with a strong capacity to incorporate and retain small exogenous molecules, e.g., pharmaceuticals. Their limited metabolic turnover provides a stable record of exposure for a long time after administration. These features make them well-suited for spatially resolved chemical analysis. This study presents the first proof-of-concept application of matrix-assisted laser desorption/ionization mass spectrometry imaging (MALDI-MSI) to visualize both endogenous metabolites and clinically relevant pharmaceuticals in situ within human dental hard tissues and jawbone. Human teeth, jawbone, and teeth with adjacent jawbone from four patients were sectioned and analyzed using high-resolution Orbitrap MSI, followed by metabolite annotation in METASPACE and confirmatory analysis. MSI successfully mapped endogenous metabolites such as arginine, phosphatidylcholine, and lysophosphatidylcholine, and revealed distinct distribution patterns of the pharmaceuticals lidocaine, bupivacaine, and chlorhexidine. Injected analgesics were predominantly localized within the tissues, whereas topically applied chlorhexidine was confined to the surface. The study demonstrates that MSI can detect retained pharmaceuticals in mineralized tissues and differentiate their spatial patterns according to route of administration. The findings highlight the value of MSI as a complement to conventional LC-MS by providing spatial context that cannot be obtained from homogenized samples. MSI can reveal the spatial distribution and accumulation of both endogenous metabolites and exogenous drugs within dental hard tissues and jawbone. Such spatial insight is valuable for understanding how pharmaceuticals interact with mineralized tissues, where they may accumulate or undergo biotransformation, and may ultimately support the development and optimization of therapeutics for dental infections such as osteomyelitis and periodontitis.

**Graphical abstract:**

**Figure:**
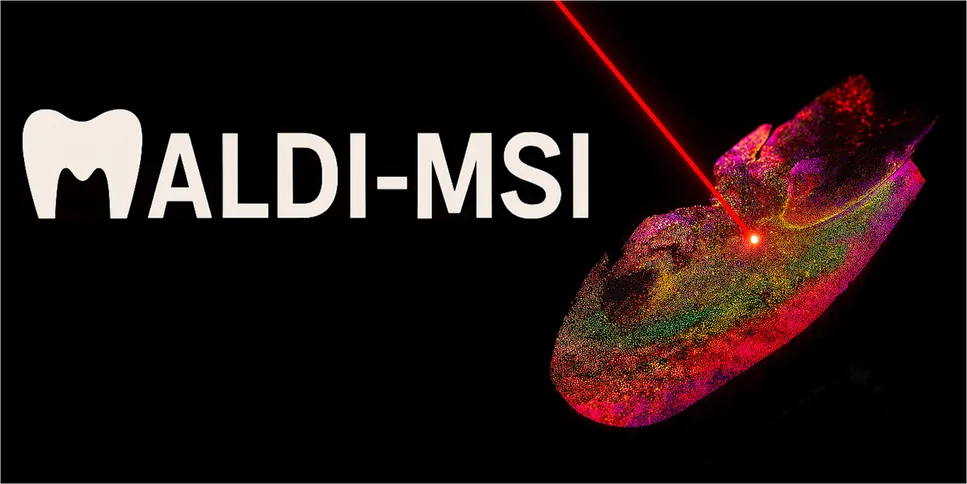

**Supplementary Information:**

The online version contains supplementary material available at https://doi.org/10.1007/s00216-026-06515-0.

## Introduction

Teeth represent a unique biological archive. Where jawbones have life-long high turnover driven by osteoblast and osteoclast activity, the dental hard tissues have very limited turnover.

The surface layers of the tooth consist of enamel covering the crown and cementum covering the root. Beneath these outer layers lies dentine, which constitutes the majority of the tooth structure and surrounds the central pulp chamber (Figure S1). Dental enamel is composed of 95–97% inorganic material (calcium hydroxyapatite crystals), compared with approximately 70% in bone and dentine, and about 50% in cementum. The remaining fractions consist of organic components and water. Collagen type 1 is part of the organic component in bone, dentine, and cementum. Bone and cementum contain numerous matrix-producing cells (i.e., osteoblasts and cementoblasts), whereas the mature dentine contains only the extensions of odontoblast processes within the dentinal tubules. The odontoblast cell bodies reside along the dentine-pulp interface. The dental pulp itself is a soft connective tissue containing blood vessels, nerves, and immune cells, and is connected to the systemic circulation and nervous system through the apical foramina [1].

Teeth and jawbone are mineralized tissues with a strong capacity to incorporate and retain small molecules due to their high content of hydroxyapatite and their limited metabolic turnover. Dental tissues may be exposed to pharmacological agents, recreational drugs, and other exogenous substances through direct contact (e.g., topical chlorhexidine or orally administered medications) or indirectly via blood circulation, salivary secretion, or diffusion across the dentoalveolar membrane at the cervical region. Experimental studies have demonstrated that a wide range of drugs can bind to, diffuse into, and become trapped within dentine during life and into enamel during tooth formation, with dentine typically accumulating significantly higher concentrations due to the life-long formation of tertiary dentine in the tooth. In vitro models have shown that drug deposition in dentine depends on physicochemical properties, exposure duration, and contact through either pulpal perfusion or the oral cavity, supporting the notion that both systemic and locally applied compounds can become incorporated into dental hard tissues over time [2]. Because dentine undergoes slow continuous remodulation by the odontoblasts, exogenous molecules can be incorporated and persist life-long after administration, providing a stable record of exposure in the dentine. In contrast, the jawbone has a higher turnover but the mineralized matrices also bind certain drugs and enable retention for long periods. This contrasts with matrices such as blood, saliva, and urine, which reflect only recent exposure. Like long hair, teeth therefore provide a long-term record of chemical incorporation, making them valuable in retrospective and forensic investigations. While there are several studies of drugs in dental biofilm (plaque) [3, 4] and in oral fluid [5, 6], the number of studies in teeth is more limited, due to the inherent invasiveness of extracting the teeth. However, previous studies have primarily relied on powdered tooth material for toxicological analysis [7–10], whereas the use of intact dental samples for spatially resolved drug detection remains unexplored. Understanding how drugs penetrate and distribute within dental tissues remains insufficiently characterized, and significant analytical challenges persist because the interactions—especially of strongly mineral-binding drugs [11]—with both the inorganic and organic components of dental hard tissues are not yet well defined.

Mass spectrometry imaging (MSI) has emerged as a powerful analytical approach capable of resolving the spatial distribution of molecular species directly within sectioned biological tissues, including plants [12], animal tissues [13], cell cultures [14], and clinical samples [15]. Applying MSI to mineralized tissues such as bone and teeth presents challenges due to their hardness and the need for decalcification to obtain thin sections [16, 17]. Nevertheless, several MSI modalities have demonstrated significant utility in research in dental hard tissues.

Laser ablation inductively coupled plasma mass spectrometry (LA-ICP-MS) has been widely used for elemental bioimaging of trace metals in teeth, achieving high spatial resolution (e.g., 50 µm) [18, 19]. This technique has revealed archived toxicological and nutritional information, including concentrations of lead, strontium, zinc, and lithium, and has provided insights into exposure chronology. A study analyzing ancient and contemporary teeth found distinct spatial patterns of elevated lead and zinc in the pulp and surface enamel, and zinc and lithium enrichment in the crown, potentially reflecting prenatal exposure [18].

Beyond elemental analysis, matrix-assisted laser desorption/ionization (MALDI) MSI has been applied to characterize the organic composition and anatomical structures of teeth. Hirano et al*.* identified characteristic signals from enamel, dentine, pulp, and periodontal ligament, and demonstrated accumulation of lysophosphatidylcholine on the root surface of teeth affected by periodontal disease, suggesting an inflammatory association [20]. MALDI-MSI has also been optimized for protein mapping in decalcified formalin-fixed paraffin-embedded mouse hemimandibles, enabling spatial identification of native proteins in developing tooth regions [16], and has been applied to archaeological teeth to visualize ancient proteins [17].

Time-of-flight secondary ion mass spectrometry (TOF-SIMS) has further contributed to the analysis of mineralized dental tissues. It has been used for quantitative imaging of dilute stable strontium labels in dentine and enamel, reconstructing zones of active mineralization with high sensitivity [21]. TOF-SIMS has also challenged traditional interpretations of incremental lines in cementum, showing that life-history events such as pregnancies correlate with reduced mineralization that may not be detected by optical microscopy alone [22]. None of the previous studies has investigated the presence or distribution of pharmaceuticals in human teeth.

Previous research highlights the need to deepen our understanding of dental microchemistry, trace element exposure, distribution of endogenous substances, and the influence of disease and life history events on tooth composition in situ. MSI is the obvious method to deepen our knowledge as it plays an increasingly important role in drug development, where it provides insight into whether drugs reach their intended sites of action, as well as where drugs accumulate and potentially undergo biotransformation. This information will be highly relevant in the understanding or even development of therapeutics for dental infections (e.g., osteomyelitis and periodontitis) or a better understanding of why some diseases develop in the jawbone (e.g., medication-related osteonecrosis of the jaws). Such insights will support the optimization of dental therapeutics, improve understanding of off-target binding in mineralized matrices, and facilitate the development of more effective drug delivery strategies for dental and bone-related conditions.

The present study aims to use MSI to characterize endogenous processes and the incorporation of exogenous substances (drugs) in dental tissues and adjacent jawbone. The original objective was to investigate the potential accumulation of bisphosphonate drugs in teeth and jawbone in patients treated with bisphosphonates. However, these strongly mineral-binding drugs [11] were not possible to detect by MSI. Instead, the study elucidated the broader applicability of MSI for mapping endogenous compounds and detecting other exogenous substances.

## Methods

### Chemicals

2,5-Dihydroxybenzoic acid (98% purity) and lidocaine were purchased from Merck KGaA (Darmstadt, Germany), and LC-MS grade methanol and water were purchased from VVW (Søborg, Denmark).

### Tooth and bone samples

The samples prepared for this study were collected from a larger cohort of patients diagnosed with medication-related osteonecrosis of the jaws (MRONJ), treated at the Department of Oral & Maxillofacial Surgery, Rigshospitalet, Copenhagen University Hospital. All patients in this study underwent surgical treatment for MRONJ following bisphosphonate therapy prescribed for their underlying conditions, including osteoporosis (patient no. 1) and malignancy (patient nos. 2 and 3). Teeth were surgically removed as isolated procedures or as part of jawbone resection. Following removal or resection, samples were collected and frozen at −76° C until sample preparation. One tooth was received from a healthy patient only exposed to local analgesics in relation to extraction of the tooth (patient no. 4). Medical data were available from the patients’ medical hospital-based electronic health records (Sundhedsplatformen). Data included information about prior and current hospital treatments as well as medications to which the patients had been exposed before and during the surgery in relation to MRONJ. After obtaining the results of the MALDI imaging, these were compared with the medical records to verify the findings of exogenous substances. Table 1 presents an overview of the samples in the study.

**Table 1:** Overview of samples in the study

Patient no.	1	2	3	4
Hard tissue	Tooth root embedded in lower jawbone	Lower jawbone	Root of tooth	Intact tooth
MRONJ	+	+	+	−
Local analgesics during the operation	Bupivacaine	Bupivacaine	Lidocaine	Lidocaine
History of local analgesics prior to the operation		Lidocaine	Lidocaine	
Figure no.	1	2	3	4

### Sample preparation

Prior to the MALDI imaging, the samples were cut in thin slices of approximately 80–100 µm thickness using a sledge microtome. As part of this process, the samples were taken directly from the freezer and embedded in polymethyl methacrylate, a two-part acrylic material, as shown in Fig. S1.

The sliced samples were thawed in a vacuum desiccator and mounted on regular microscope glass slides using double-sided graphite tape (Electron Microscopy Sciences, Hatfield, PA, USA). The samples were coated with matrix using a pneumatic sprayer as previously described [23]. Three hundred microliters of a 30 mg/mL solution of 2,5-dihydroxybenzoic acid (DHB) in MeOH/H_2_O (70:30) was sprayed at a flow rate of 30 µL/min with a nebulized gas pressure of 2 bar and the sample rotating at 150 rpm. The homogeneity of the matrix layer was controlled by inspection using an Olympus BH-2 microscope with reflected light from an external light source.

### MALDI-MSI

MALDI-MSI analysis was performed on a Thermo QExactive Orbitrap mass spectrometer (Thermo Fisher Scientific GmbH, Bremen, Germany) equipped with an AP-SMALDI5 ion source (TransMIT GmbH, Giessen, Germany). The analysis was carried out in positive ionization mode at a mass resolution of 140,000@*m*/*z*200. The scan range was set to *m*/*z* 125–875, and a DHB matrix peak was used as a lock mass to ensure a mass accuracy of ≤ 2 ppm. The samples were imaged with a pixel size of 50 µm or 70 µm in line-scan mode, using a laser spot size of approx. 20 µm, thus preventing any instances of oversampling.

Raw data was converted to imzML using the RAW+UDP to IMZML software (v1.6R170; TransMIT GmbH, Giessen, Germany). The imzML files were uploaded to the METASPACE online metabolite annotation platform (metaspace2020.org) for further analysis. Images were generated in MSiReader 1.02 [24] using a mass tolerance of ± 2 ppm and no normalization of intensities. The intensity color scale in the images is normalized individually for each image, and the intensities as read by the colors in the images are thus only comparable within one single image and not across different images.

The results were inspected in the METASPACE online annotation platform. Upon upload of data, METASPACE automatically generates a number of images (depending on the type of sample and the quality of the data, up to several hundred images); for each image, a molecular formula is assigned, derived from accurate mass analysis and the isotope pattern, and through database searches, a number of compound identities are proposed [25]. Importantly, the identifications in METASPACE should be considered tentative, as they are based solely on accurate mass analysis and isotope patterns, leaving open the possibility of interferences from isomeric and closely spaced isobaric compounds.

### DESI-MS analysis

For confirmation of the presence of lidocaine in the samples, MS/MS analysis was conducted on a Thermo LTQ XL linear ion trap mass spectrometer, equipped with a custom-built DESI-MSI ion source described in detail elsewhere [26]. The spray solvent, a methanol and water mixture (95:5), was delivered at a flow rate of 5 µL/min, and the nebulizer gas pressure was 7 bar.

## Results and discussion

**Fig. 1:**
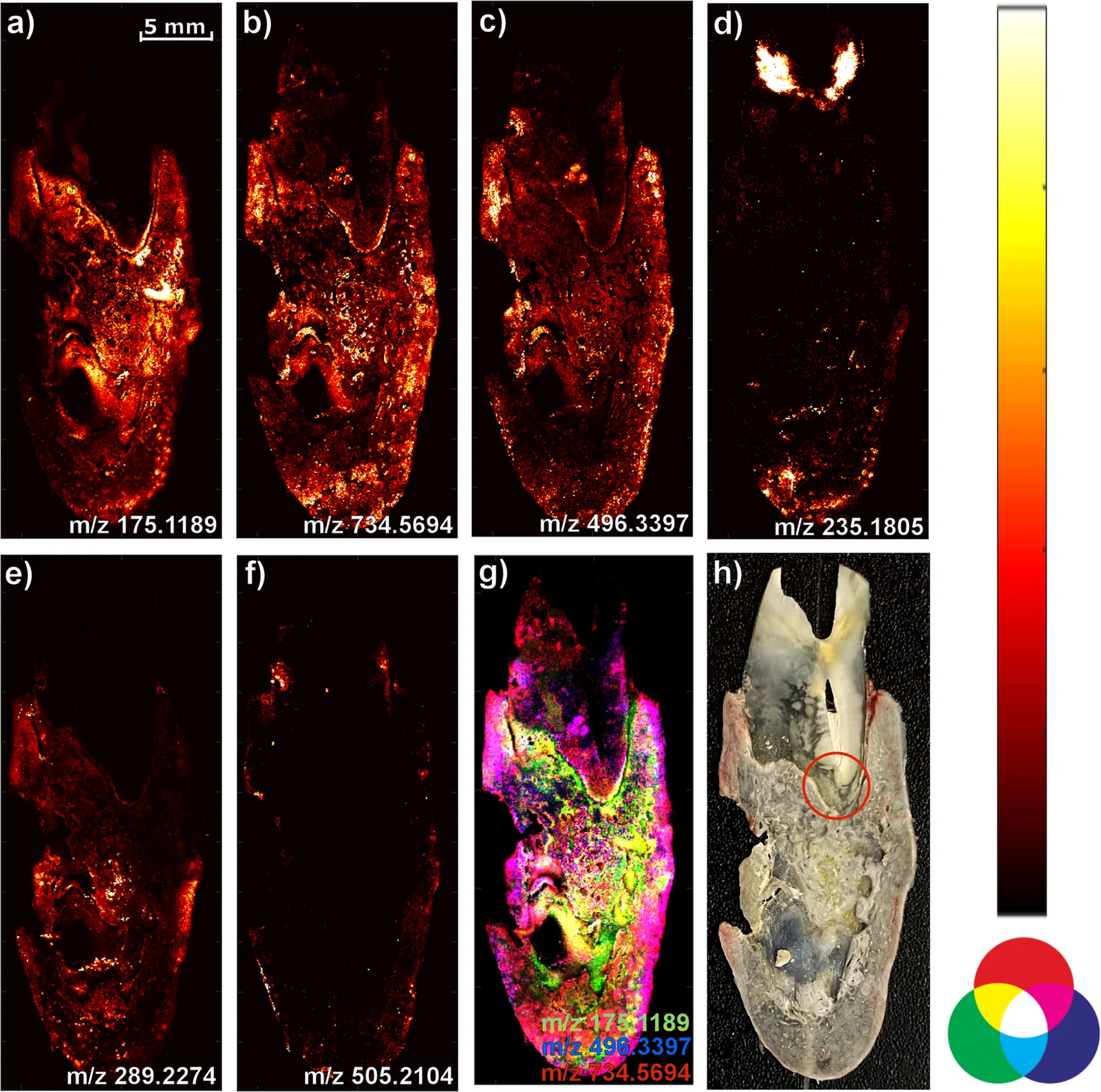
MALDI MS images of a tooth root embedded in the adjacent lower jawbone (patient no. 1). **a** Arginine ([M+H]^+^, *m*/*z* 175.1189). **b** Phosphatidylcholine (32:0) ([M+H]^+^, *m*/*z* 734.5694). **c** Lysophosphatidylcholine (16:0) ([M+H]^+^, *m*/*z* 496.3397). **d** Lidocaine ([M+H]^+^, *m*/*z* 235.1805). **e** Bupivacaine ([M+H]^+^, *m*/*z* 289.2274). **f** Chlorhexidine ([M+H]^+^, *m*/*z* 505.2104). **g** Overlay of arginine (green), lysophosphatidylcholine (16:0) (blue), and phosphatidylcholine (32:0) (red). **h** Photo. The red circle highlights the interface between the tooth cementum and the bone

**Fig. 2:**
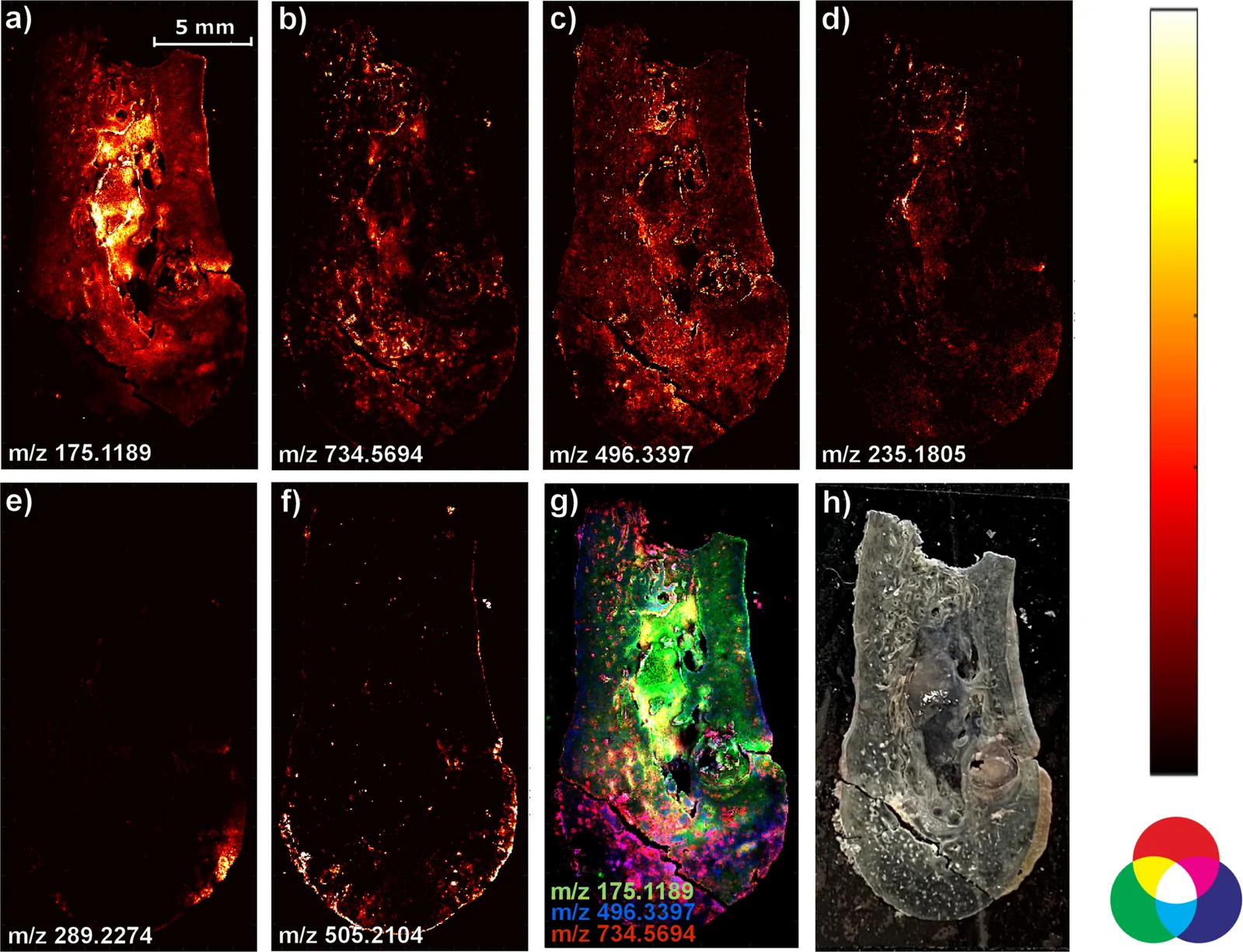
MALDI MS images of lower jawbone (patient no. 2). **a** Arginine ([M+H]^+^, *m*/*z* 175.1189). **b** Phosphatidylcholine (32:0) ([M+H]^+^, *m*/*z* 734.5694). **c** Lysophosphatidylcholine (16:0) ([M+H]^+^, *m*/*z* 496.3397). **d** Lidocaine ([M+H]^+^, *m*/*z* 235.1805). **e** Bupivacaine ([M+H]^+^, *m*/*z* 289.2274). **f** Chlorhexidine ([M+H]^+^, *m*/*z* 505.2104). **g** Overlay of arginine (green), lysophosphatidylcholine (16:0) (blue), and phosphatidylcholine (32:0) (red). **h** Photo

**Fig. 3:**
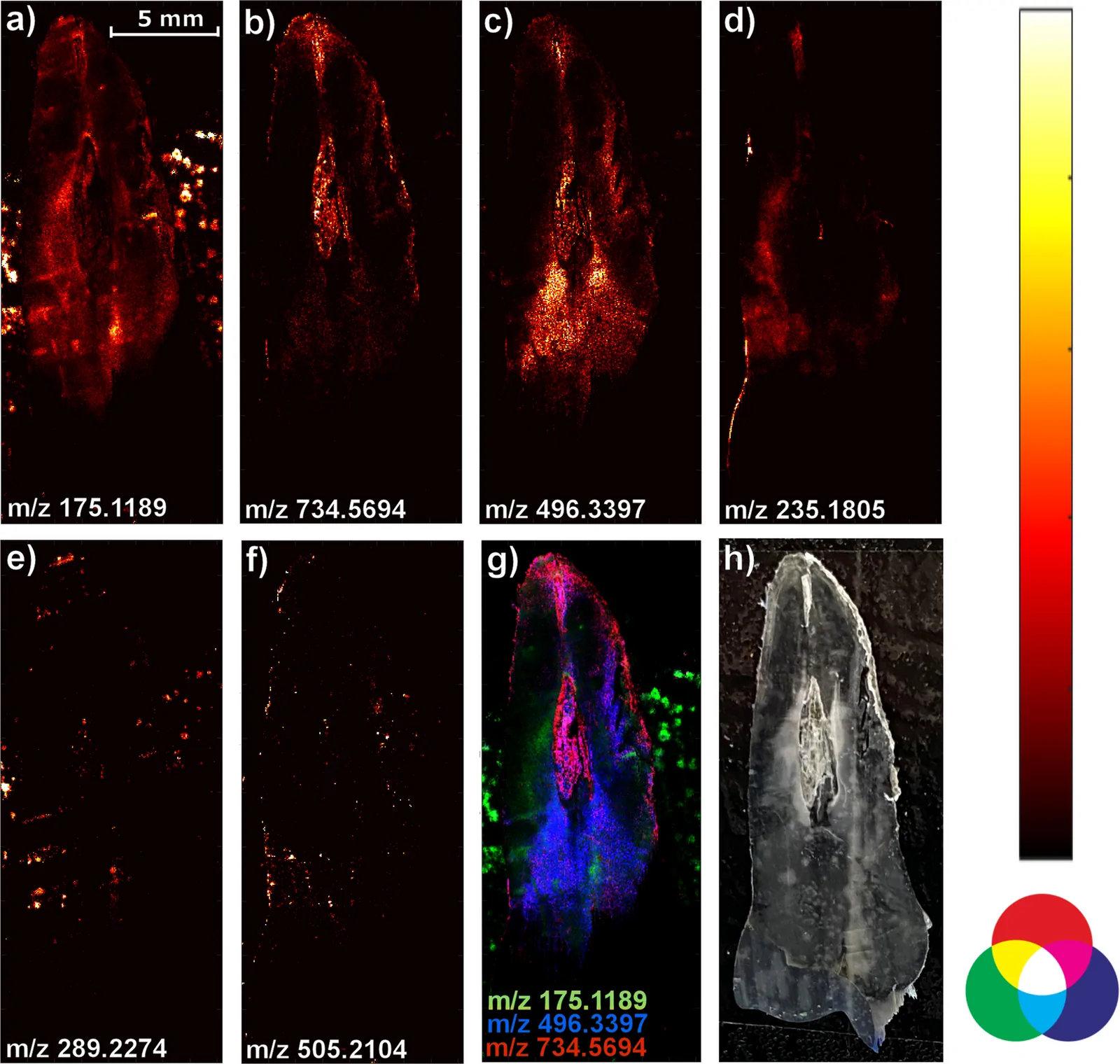
MALDI MS images of the root of a molar from the upper jaw (patient no. 3). **a** Arginine ([M+H]^+^, *m*/*z* 175.1189). **b** Phosphatidylcholine (32:0) ([M+H]^+^, *m*/*z* 734.5694). **c** Lysophosphatidylcholine (16:0) ([M+H]^+^, *m*/*z* 496.3397). **d** Lidocaine ([M+H]^+^, *m*/*z* 235.1805). **e** Bupivacaine ([M+H]^+^, *m*/*z* 289.2274). **f** Chlorhexidine ([M+H]^+^, *m*/*z* 505.2104). **g** Overlay of arginine (green), lysophosphatidylcholine (16:0) (blue), and phosphatidylcholine (32:0) (red). **h** Photo

**Fig. 4:**
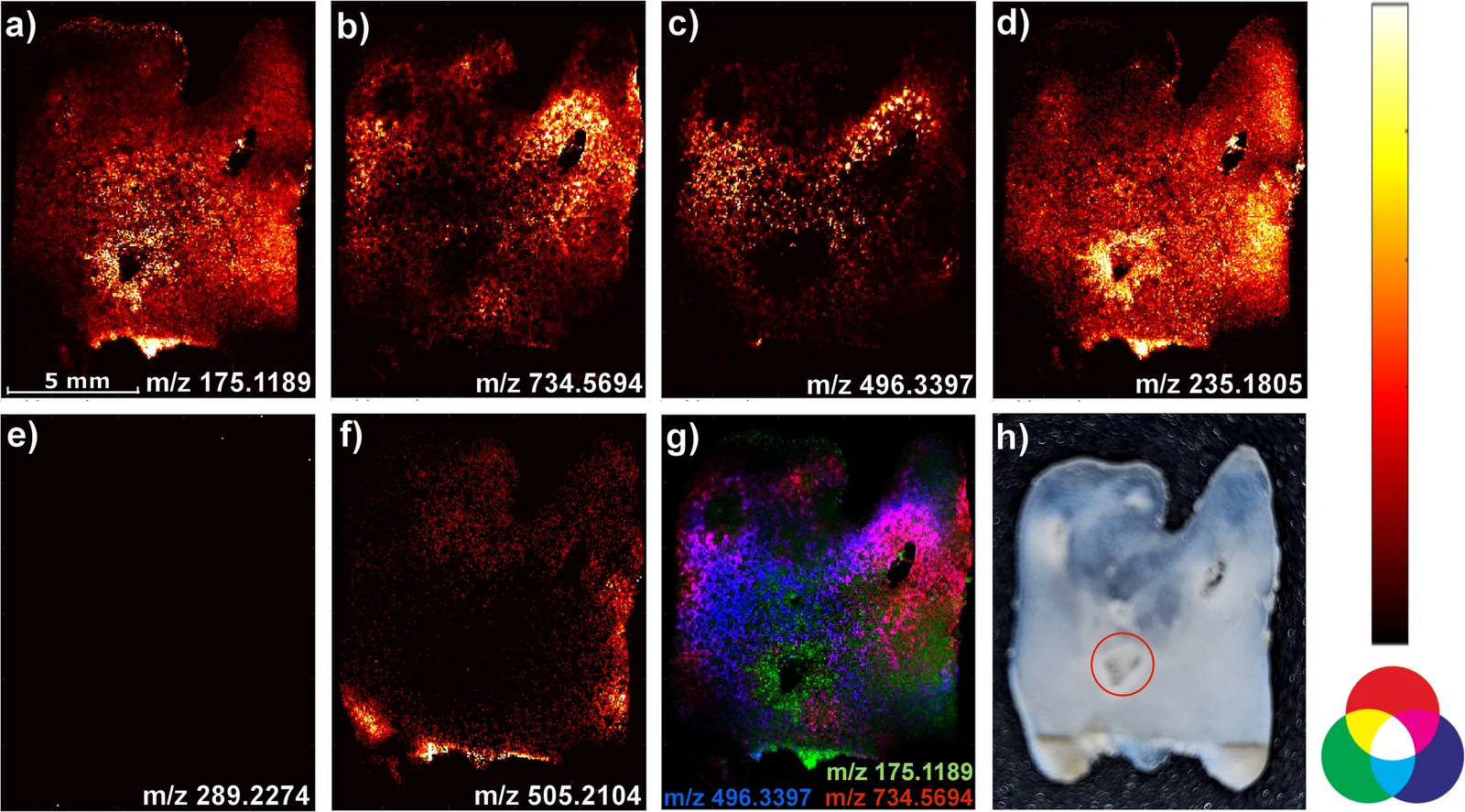
MALDI MS images of the third molar in the upper jaw (patient no. 4). **a** Arginine ([M+H]^+^, *m*/*z* 175.1189). **b** Phosphatidylcholine (32:0) ([M+H]^+^, *m*/*z* 734.5694). **c** Lysophosphatidylcholine (16:0) ([M+H]^+^, *m*/*z* 496.3397). **d** Lidocaine ([M+H]^+^, *m*/*z* 235.1805). **e** Bupivacaine ([M+H]^+^, *m*/*z* 289.2274). **f** Chlorhexidine ([M+H]^+^, *m*/*z* 505.2104). **g** Overlay of arginine (green), lysophosphatidylcholine (16:0) (blue), and phosphatidylcholine (32:0) (red). **h** Photo. The red circle highlights the pulp horn

A number of samples from MRONJ patients were imaged, and selected images of the samples from the cohort are presented in Figs. 1, 2, and 3. Figure 1 shows a tooth root in the adjacent jawbone, Fig. 2 shows a section of jawbone, and Fig. 3 shows the root of a tooth. In addition, an imaging experiment was conducted on a tooth extracted from a healthy patient. Results from this experiment are presented in Fig. 4.

Data for all four samples have been uploaded to the METASPACE platform where they can be freely accessed. In general, compared to MSI data from soft tissues, which are abundant in METASPACE, the number of compound annotations is significantly lower for hard tissues than for, e.g., brain. In addition, the number of lipids—constituting a large fraction of the annotations in soft tissues—is markedly reduced in these samples. This is not surprising, as a relatively large fraction of tooth and bone consists of inorganic material and mineral-bound compounds, which are not readily accessible for detection by MALDI-MSI. However, during browsing of the data in METASPACE, annotations appeared for well-known compounds like lidocaine and chlorhexidine, which are known from their use in the oral cavity by dentists and patients. When this became known, information was searched in the patient records for other drugs administered to the patients as part of their treatments, and a control sample (a tooth) from a healthy person was also analyzed.

As mentioned in the Experimental section, the samples—hard, undecalcified tissues—were sliced using a sledge microtome. Decalcification, which would have made sectioning easier, was not performed to avoid the risk of analyte delocalization prior to imaging. In other MSI studies of hard tissues like bone [27] or whole mice with teeth and bone [28], sectioning was performed on a cryo-microtome equipped with a tungsten carbide blade using tape for collection of the tissue sections. A similar approach might also have worked for the tissues in this study.

### Endogenous substances of the hard oral tissues

As mentioned, compared to the datasets for soft tissues found in METASPACE, only a few endogenous compounds were detected in the oral hard tissues. Of these, we focus here on arginine, phosphatidylcholine (32:0), and lysophosphatidylcholine (16:0), which are shown in all four figures. Arginine, glutamic and aspartic acid, lysine, hydroxylysine, and histidine are charged amino acid residues in collagen 1. The collagen template of charged amino acid residues might be involved in the ion binding and subsequently be important for the formation of calcium hydroxyapatite crystals in the mineralized tissue [29]. Lysophosphatidylcholine (LysoPC) and phosphatidylcholine (PC) are two of several choline-phospholipids described as being part of the phospholipids associated with teeth and bone [20, 30] where they may be localized to the dental pulp and cell components present in the jawbone, dentine and the cementum. A priori, strong signals of arginine and phosphatidylcholine (PC) are expected in vital tissue, with low signals in necrotic tissue, whereas elevated LysoPC signals are expected in areas of inflammation or necrosis. Accordingly, strong arginine and PC signals are anticipated in vital cells, such as osteoblasts, cementoblasts, and odontoblasts. In vital acellular dentine, an arginine signal is also expected, albeit weaker than in cellular regions.

Arginine (Figs. 1a, 2a, 3a, and 4a, and green color in RGB overlays in Figs. 1g, 2g, 3g, and 4g) is an endogenous semi-essential amino acid involved in numerous biosynthetic pathways along with the metabolism of collagen 1 [31] and is clearly detected in all four samples. Arginine serves as a caries-preventive effect in a few toothpastes [32], but it is unlikely that this is the reason why we detect arginine. Bright arginine signal was detected in the interface of the cementum and the jawbone in patient no. 1 (Fig. 1a), in the jawbone of patient no. 2 (Fig. 2a), throughout most of the root of patient no. 3 (Fig. 3a, disregarding artifact signal from outside the tissue, possibly related to the mounting double-sided graphite tape where dust from the slicing may have attached), and along the dentine/pulp interface at the pulp horn in patient no. 4 (Fig. 4a).

The presence of arginine suggests collagen metabolism or catabolism. Interpretation can be done when evaluated together with the signals of phospholipids like phosphatidylcholine (32:0) (PC, Figs. 1b, 2b, 3b, and 4b, red color in RGB overlays in Figs. 1g, 2g, 3g, and 4g). PC is part of cell membranes and known to be incorporated between collagen fibers in the dentine [33]. Arginine together with PC suggests collagen metabolism (green, red, or yellow in Figs. 1g, 2g, 3g, and 4g). Lysophosphatidylcholine (16:0) (LysoPC, Figs. 1c, 2c, 3c, and 4c, blue color in RGB overlays in Figs. 1g, 2g, 3g, and 4g) has been associated with site of inflammation and in dentine in relation to the site of periodontal disease [20]. LysoPC was detected centrally of the extracted teeth of patient nos. 3 and 4 and together with PC in the outermost part of the compact bone of patient no. 1. LysoPC alone (blue) or together with PC suggests inflammation.

### Exogeneous substances in the hard tissues

Annotations in the data in METASPACE suggested the presence of the dental pharmaceuticals lidocaine (Figs. 1d, 2d, 3d, and 4d), bupivacaine (Figs. 1e, 2e, 3e, and 4e), and chlorhexidine (Figs. 1f, 2f, 3f, and 4f) in some of the samples, and subsequently the compounds—including other medications from the medical records—were searched for manually in data from all the imaged samples. When browsing the data in METASPACE, the compounds do not readily show up in all samples with the false discovery rate (FDR) in its lowest setting of 5%, but they appear—along with numerous other compounds—when the FDR is increased to higher levels.

Although we had not specifically expected to detect lidocaine, its presence in all four samples is not surprising, given that it is the most commonly used local anesthetic in dental practice. If any drug were to be detected, lidocaine would be a plausible candidate. For confirmation of its detection, MS/MS analysis was performed by DESI-MS on a linear ion trap mass spectrometer, using the sample from Fig. 1 and a lidocaine standard deposited on a porous PTFE surface (results shown in Supplementary Information). The MS/MS fragment of lidocaine at *m*/*z* 86 was clearly observed in both the standard and the tooth sample, confirming the presence of lidocaine in the tooth and bone.

Bright signals of lidocaine were found in jawbone samples from both patients exposed to lidocaine during surgery (patient nos. 3 and 4) and in the one patient with a history of prior exposure to lidocaine (patient no. 2). Lidocaine was detected in the bone sample from patient no. 1, despite the absence of hospital records documenting exposure to lidocaine; however, lidocaine is the most widely used local anesthetic agent in dental practice [34]. This suggests that lidocaine stays for a prolonged time in the dental tissues after exposure.

Bupivacaine—a less frequently used local analgesic, but with a longer-lasting effect—was detected throughout the jawbone of patient no. 1 (Fig. 1e) and in the outer parts of patient no. 2 (Fig. 2e), but only as a weak, scattered signal inside and outside the sample in Fig. 3e. The presence of bupivacaine was not confirmed by MS/MS analysis. However, it is known from the medical record (Table 1) that the bupivacaine was used in the treatment of the two patients. Furthermore, a control sample (a tooth) was analyzed from a patient who had not been treated with bupivacaine. The complete absence of bupivacaine signal from this sample (Fig. 4e) confirms that the bupivacaine signal in Figs. 1e and 2e is not due to interference with an endogenous compound.

Chlorhexidine, a commonly used antimicrobial antiseptic in oral mouthwash products, was reliably detected in the samples from patients 1, 2, and 4, notably primarily on the edge of the samples, which is in good agreement with chlorhexidine being absorbed topically through mouthwash. In contrast, lidocaine and bupivacaine being administered by injection and arriving to the tissues via the bloodstream were to a greater extent found in the inner parts of the tooth and bone.

No MS/MS analysis was conducted of chlorhexidine, but with its molecular formula of C_22_H_30_Cl_2_N_10_, it has a characteristic isotope pattern originating from its two chlorine atoms. Figure S4 shows the mass spectrum around the *m*/*z* 505 peak of chlorhexidine, which has great similarity to the simulated mass spectrum of chlorhexidine shown in Figure S5, reproducing all five peaks of the isotope pattern with their correct relative intensities. To further confirm the presence of chlorhexidine, images were generated from the data presented in Fig. 1 of chlorhexidine based on the signals from the ^35^Cl^35^Cl version of the molecule (*m*/*z* 505.2104), the ^35^Cl^37^Cl version (*m*/*z* 507.2075), and the ^37^Cl^37^Cl version (*m*/*z* 509.2045). As seen in Figure S6, the identical distributions of the three corresponding *m*/*z* values, which all fit with chlorhexidine, are a very strong indication that the antiseptic was detected in the teeth and bone.

### Analytical considerations on non-targeted vs. targeted MSI analysis

From an analytical standpoint, one of the most interesting aspects of the present study is the use of the metabolite annotation platform METASPACE, which enabled extensive exploration of the acquired MSI data. Initially, the study aimed to investigate bisphosphonate drugs in situ in teeth and jawbone from patients with malignancy or osteoporosis [35]. However, bisphosphonates could not be detected by MALDI-MSI, most likely due to their strong binding to calcium in hydroxyapatite crystals [36]. These compounds have previously been detected by LC-MS/MS following phosphoric acid treatment of bone and subsequent derivatization to enable retention of these highly polar compounds on reversed-phase HPLC columns [37]. However, such pretreatment is not readily feasible in an MSI context.

Upon uploading the raw MSI data to METASPACE, the platform generated numerous ion images together with tentative annotations based on accurate mass measurements from the Orbitrap mass spectrometer. Inspection of these images revealed three exogenous compounds—lidocaine, bupivacaine, and chlorhexidine—alongside endogenous metabolites. This unexpected observation highlights the analytical power of non‑targeted high‑resolution mass spectrometry in imaging applications. In particular, the combination with advanced data analysis platforms such as METASPACE enables the extraction of meaningful information from complex MSI datasets through statistical scoring and FDR‑controlled annotation.

One important caveat, however, is that METASPACE annotations should not be accepted uncritically. The platform relies exclusively on full‑scan MS1 data and reports annotations at the level of molecular formulas; the associated false discovery rate (FDR) values therefore apply to the formulas only, not to the proposed compound names. In cases where several isomers share the same elemental composition, METASPACE cannot determine which structure is most likely. Consequently, additional sources of information—biological plausibility, clinical metadata, comparison with control samples, characteristic isotope patterns, or MS/MS data—should be considered when assigning compound identities. In the present study, the identification of the three drugs was supported not only by the initial METASPACE annotations but also by patient records, comparison to untreated control tissue, distinctive isotopic signatures, and, in the case of lidocaine, confirmatory MS/MS analysis. Such complementary evidence increases the overall confidence in the reported compound annotations.

The present results were obtained using non-targeted MSI in full-scan mode on an Orbitrap mass spectrometer equipped with an AP-SMALDI5 ion source. This MSI configuration offers sensitivity comparable to many other Orbitrap-based or QTOF-based MSI platforms currently in use. In contrast, recently developed DESI-MSI systems optimized for targeted imaging, employing next-generation DESI sprayers in combination with triple-quadrupole mass spectrometers, have been reported to provide substantially increased sensitivity [38].

Therefore, targeted MSI approaches have the potential to enable the detection of a substantially larger number of pharmaceutical compounds than demonstrated in the present study. In particular, drugs present at very low concentrations—such as compounds administered years prior to sampling—may be accessible using highly sensitive targeted acquisition strategies. However, such approaches rely on prior knowledge of the patient’s medication history, as the analytes of interest must be predefined to be selectively targeted during data acquisition.

Although the number of samples in this study is limited, the results illustrate only the very surface of what is possible. With advances in targeted, ultra‑sensitive MSI methods, it is likely that even drugs administered long before sampling could be detected at meaningful spatial resolution. Moreover, combining non‑targeted LC‑MS of powdered dental material—which may offer superior sensitivity for initial screening—with subsequent targeted MSI of selected compounds represents a powerful future workflow for comprehensive chemical characterization of teeth.

## Conclusion

In conclusion, this study provides the first proof‑of‑concept demonstration that mass spectrometry imaging (MSI) can be used to visualize the spatial distribution of drugs within mineralized dental tissues, including teeth and jawbone. We show that MSI is capable of detecting both endogenous metabolites and exogenous compounds administered during surgical procedures, and we reveal clear differences between the spatial patterns of injected versus topically applied drugs. These findings highlight the value of MSI as a complementary approach to conventional LC‑MS, as MSI uniquely preserves the tissue context and allows direct visualization of compound localization within the microanatomy of mineralized tissues.

Overall, this work demonstrates the potential of MSI to investigate drug uptake and distribution in teeth, setting the stage for future studies aimed at understanding tissue remodeling, incorporation of exogenous substances, tissue reaction towards dental diseases, and development of new therapeutics for dental and skeletal disorders in the jaws. As the methodology continues to evolve, MSI is well positioned to become a key tool for elucidating the molecular processes that govern both healthy and diseased mineralized tissues.

## Supplementary Information

Below is the link to the electronic supplementary material.

Supplementary file1 (PDF 463 KB)

## Data Availability

The raw mass spectrometry imaging data generated in this study have been deposited in the METASPACE platform and can be accessed as described in the “Associated content” section. Processed data, annotations, figures, and additional supporting information are provided within the manuscript and Supplementary Information. Any additional data are available from the corresponding author upon reasonable request.

## Declarations

### Associated content

Raw data from the study in the form of imzML files have been uploaded to the METASPACE annotation platform from where they can be freely accessed via the following link: https://metaspace2020.org/project/dental-hard-tissues
