# Supplementary material for: In situ MALDI mass spectrometry imaging of pharmaceuticals and metabolites in human teeth and jawbone
Source: Anal Bioanal Chem. 2026 Apr 23;418(20):6657–67. doi: 10.1007/s00216-026-06515-0 (PMC13619759; doi:10.1007/s00216-026-06515-0)
Supplement: Supplementary file 1 [file 216_2026_6515_MOESM1_ESM.pdf]

In situ MALDI mass spectrometry imaging of pharmaceuticals and metabolites in human teeth and jawbone

Supplementary information

Hameda Amin [4], Marcus Daniel Brandbjerg Bohn Lorensen [1], Harald Severin Hansen [2], Azam Bakhshandeh [5], Thomas Kofod [4], Sanne Werner Moeller Andersen [4], Camilla Kragelund [3] and Christian Janfelt [1].

1) Department of Pharmacy, Faculty of Health and Medical Sciences, University of Copenhagen, Universitetsparken 2, 2100 Copenhagen, Denmark.
2) Department of Drug Design and Pharmacology, Faculty of Health and Medical Sciences, University of Copenhagen, Universitetsparken 2, 2100 Copenhagen, Denmark.
3) Faculty of Odontology, Department of Oral Pathology, Malmö University, Malmö, Sweden.
4) Department of Oral & Maxillofacial Surgery, Copenhagen University Hospital (Rigshospitalet), Copenhagen, Denmark.
5) Department of Odontology, Faculty of Health and Medical Sciences, University of Copenhagen, Noerre Alle 20, 2200 Copenhagen, Denmark.

Correspondence to:
Christian Janfelt, Tel.: +45 35 33 65 57; fax: +45 35 33 60 30
E-mail address: christian.janfelt@sund.ku.dk

Submitted to *Analytical Bioanalytical Chemistry*, January 2026.

Contents

Sectioning of teeth and bone, tooth anatomy 2
Confirmation of lidocaine in teeth 2
Confirmation of chlorhexidine in jawbone 4

Sectioning of teeth and bone, tooth anatomy

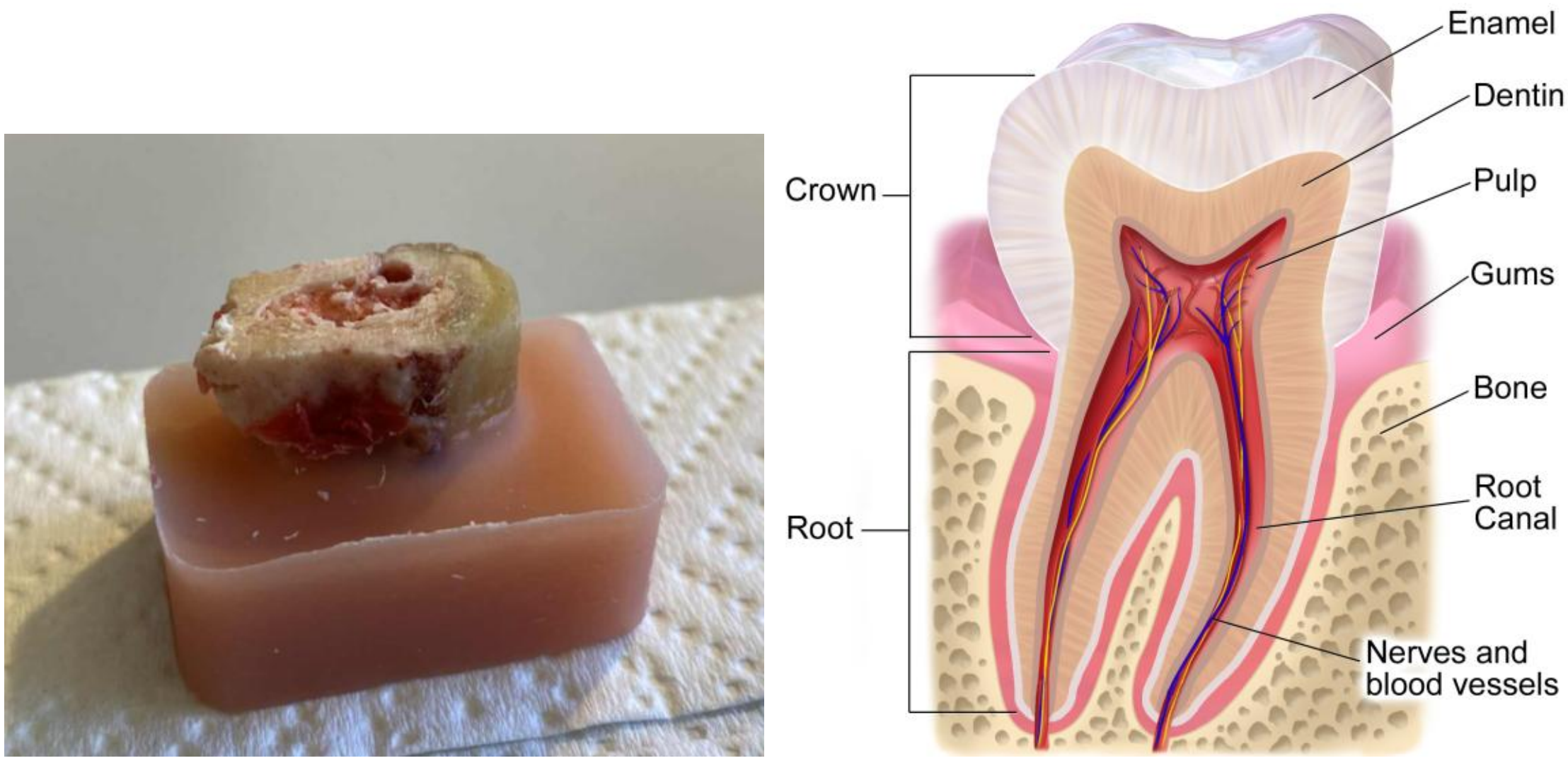

Figure S1: *Left: Embedding of the tooth and bone in acrylic material prior to sectioning on a sledge microtome. Right: The anatomy of the tooth (figure from Blausen.com staff (2014). "Medical gallery of Blausen Medical 2014". WikiJournal of Medicine 1 (2). DOI:10.15347/wjm/2014.010. ISSN 2002-4436.)*

Confirmation of lidocaine in teeth

For confirmation of the presence of lidocaine in the samples, MS/MS analysis was conducted in positive ion mode on a Thermo LTQ XL linear ion trap mass spectrometer, equipped with a custom-built DESI-MSI ion source. The spray solvents, a methanol and water mixture (95:5), was a delivered at a flow rate of 5 µL/min and the nebulizer gas pressure was 7 bar.

MS/MS spectra of lidocaine were acquired from a lidocaine standard (1 mg/mL dissolved in methanol), deposited and smeared in a very thin layer on porous PFTE for evaporation, as seen in Figure S2. Importantly, the spectra were not observed on areas of the PTFE surface where lidocaine was not deposited.

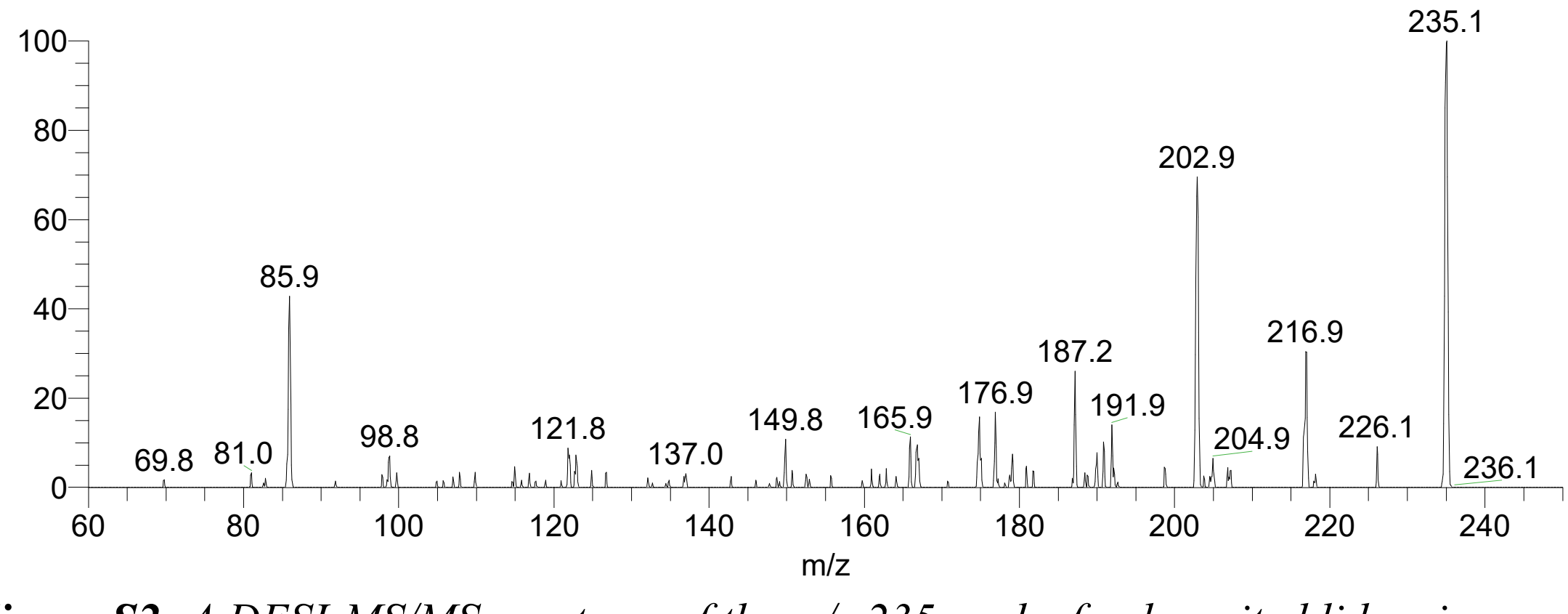

Figure S2: *A DESI-MS/MS spectrum of the m/z 235 peak of a deposited lidocaine standard solution.*

Then, a section of the tooth in Figure 3 was analyzed in MS/MS, isolating and fragmenting the *m/z* 235 ion of protonated lidocaine, as seen in Figure S2:

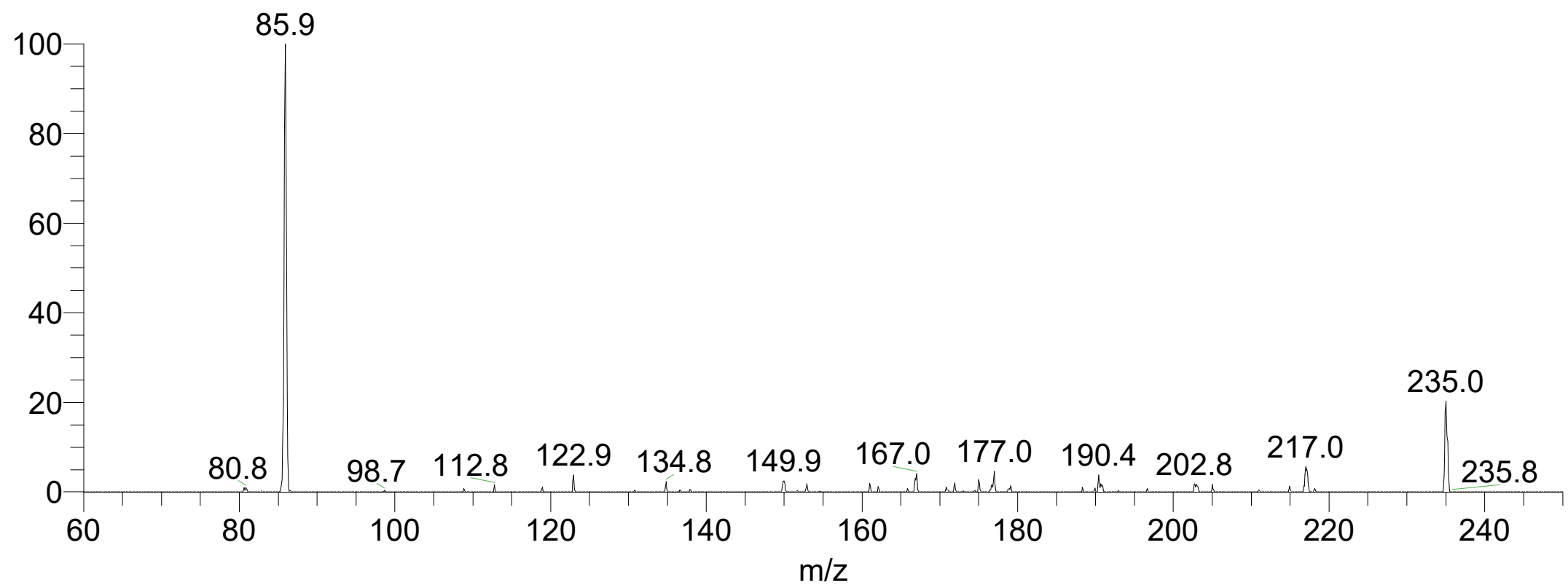

Figure S3: *A DESI-MS/MS spectrum of the m/z 235 peak of a slice of the tooth shown in Figure 3. All major peaks in the spectrum are also observed in the MS/MS spectrum of the lidocaine standard in Figure S2.*

Similarities in the two MS/MS spectra confirm that lidocaine was present in the tooth analyzed for Figure 3, and that lidocaine contributes to the *m/z* 235.1805 signal in the image.

Confirmation of chlorhexidine in jawbone

Mass spectra were extracted from the raw data from Figure 2 of jawbone from some of the pixels with the highest intensities of m/z 505.2104, tentatively assigned to chlorhexidine. Figure S4 shows the part of the mass spectrum from the sample.

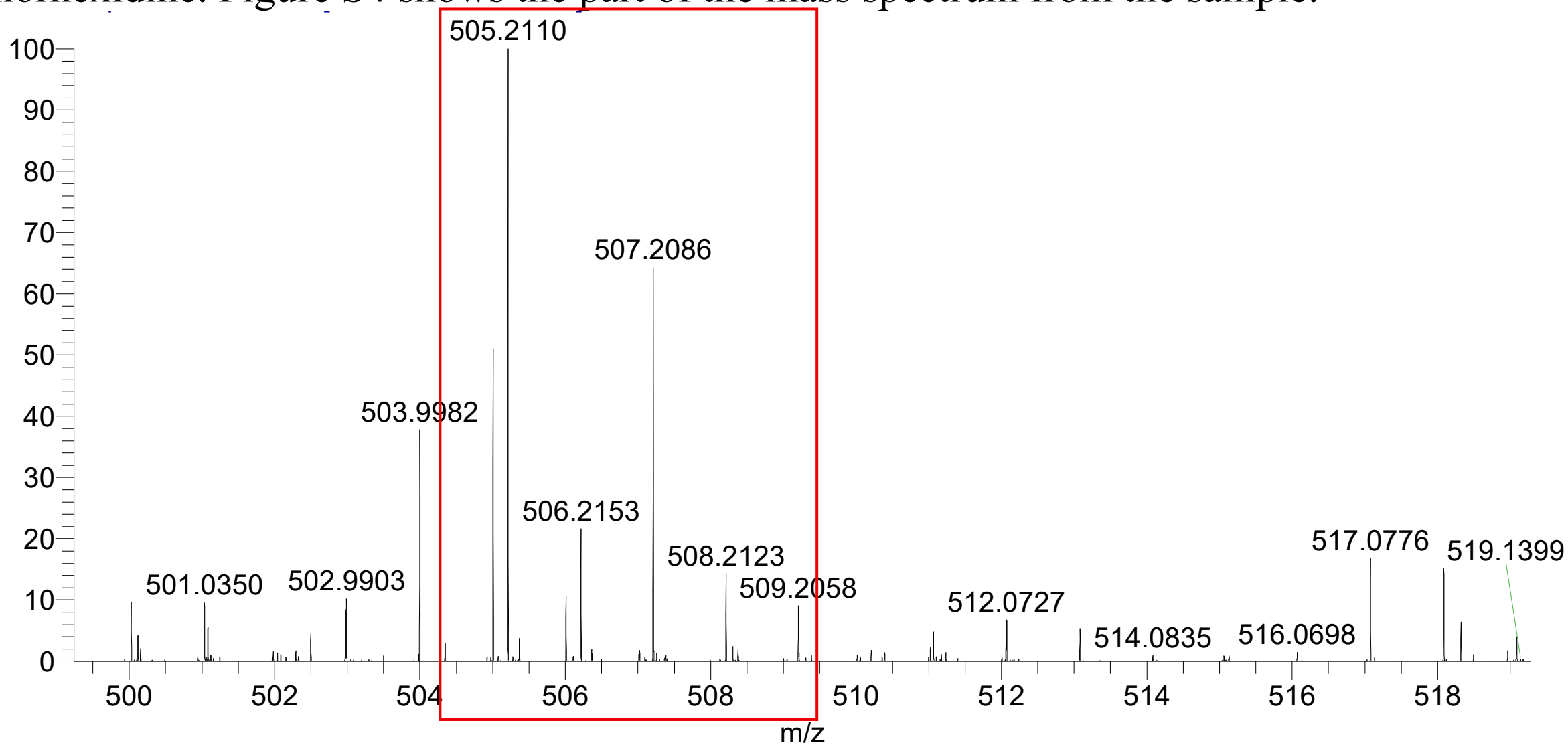

Figure S4: *Mass spectrum extract from pixels in the image from Figure 2. The red box highlights the signature isotope pattern of chlorhexidine.*

For comparison, the mass spectrum of protonated chlorhexidine ($C_{22}H_{30}Cl_2N_{10}$) was simulated in the XCalibur software, as shown in Figure S4.

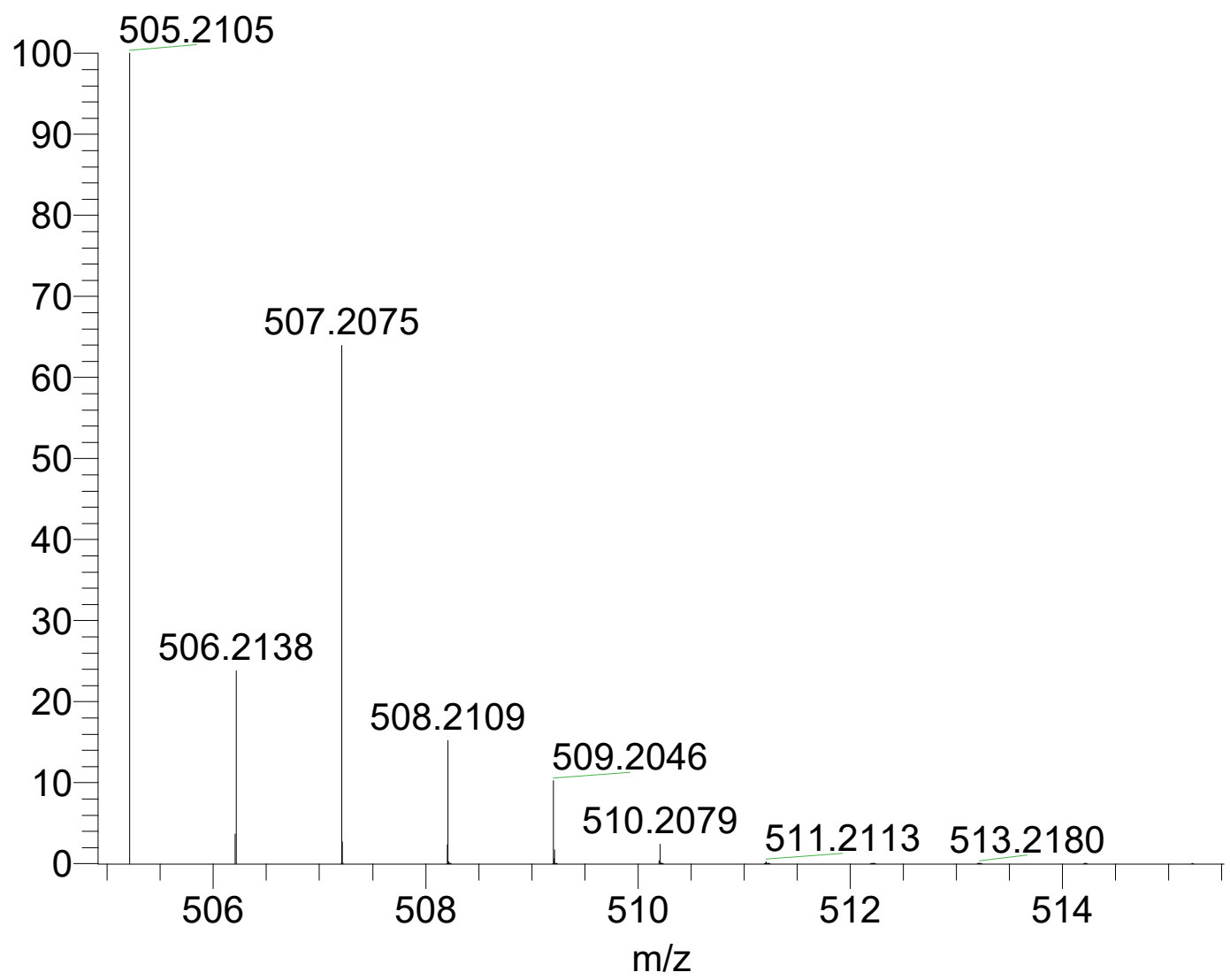

Figure S5: *Simulated mass spectrum of protonated chlorhexidine ($C_{22}H_{30}Cl_2N_{10}$), showing the characteristic isotope pattern of two chlorine atoms.*

A seen by comparing Figure S4 and S5, as the isotope peaks of chlorhexidine are found in the tooth within a mass accuracy of ± 1 mDa, providing the evidence of the presence of chlorhexidine (or an isomer of it, however unlikely that may seem).

In addition, for the same sample, images were created of *m/z* values of the major isotope peaks, as seen in Figure S6.

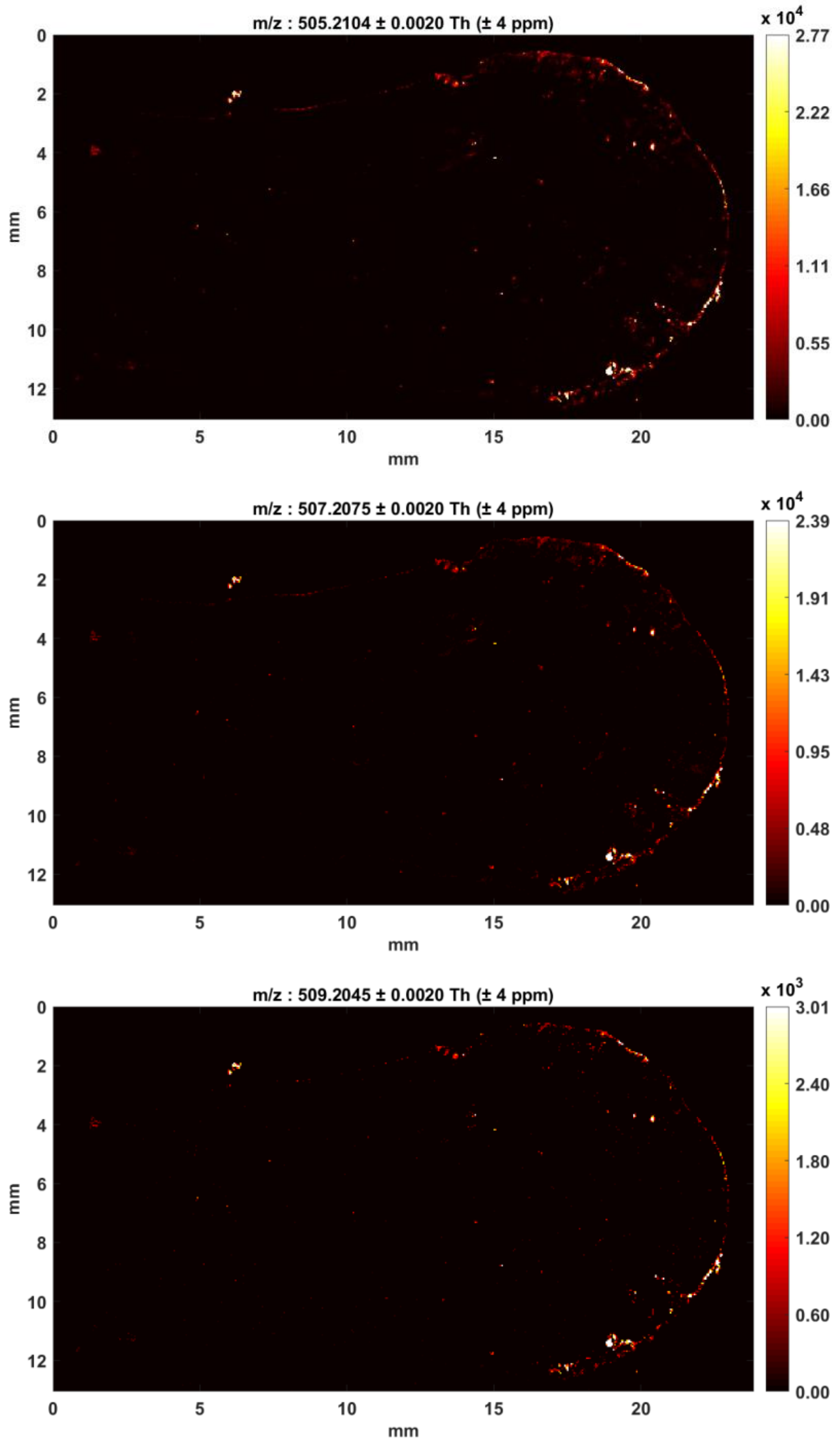

Figure S6. *Images of the jawbone from Figure 2, extracted for each of the three major peaks in the isotope pattern of chlorhexidine. The enhance the brightness of the images for clearer assessment of identical distributions, the images were generated with a bin width of ± 4 ppm, and the color scales were brightened individually.*

For the chlorhexidine signal in all samples of the studies, similar images could be generated for the three major peaks in the isotope pattern (data not shown).
